# Bilateral testicular self-castration due to cannabis abuse: a case report

**DOI:** 10.1186/1752-1947-5-404

**Published:** 2011-08-23

**Authors:** Mustapha Ahsaini, Fadl Tazi, Abdelhak Khalouk, Karim Lahlaidi, Abderahim Bouazzaoui, Roos E Stuurman-Wieringa, Mohammed Jamal Elfassi, My Hassan Farih

**Affiliations:** 1 Department of Urology, Hospital University Center Hassan II, 30000 Fez, Morocco; 2 Department of Anesthesia and Intensive Care Unit, Hospital University Center Hassan II, 30000 Fez, Morocco; 3 Department of Urology, Academic Medical Center, PO Box 22660, 1100 DD, Amsterdam, The Netherlands

## Abstract

**Introduction:**

The self-mutilating patient is an unusual psychiatric presentation in the emergency room. Nonetheless, serious underlying psychiatric pathology and drug abuse are important background risk factors. A careful stepwise approach in the emergency room is essential, although the prognosis, follow-up, and eventual rehabilitation can be problematic.

We present a unique and original case of bilateral self-castration caused by cannabis abuse.

**Case Presentation:**

We report a case of a 40-year-old Berber man, who was presented to our emergency room with externalization of both testes using his long fingernails, associated with hemodynamic shock. After stabilization of his state, our patient was admitted to the operating room where hemostasis was achieved.

**Conclusion:**

The clinical characteristics of self-mutilation are manifold and there is a lack of agreement about its etiology. The complex behavior associated with drug abuse may be one cause of self-mutilation. Dysfunction of the inhibitory brain circuitry caused by substance abuse could explain why this cannabis-addicted patient lost control and self-mutilated. To the best of our knowledge, this is the first case report which presents an association between self-castration and cannabis abuse.

## Introduction

Self-inflicted testicular injuries are an uncommon phenomenon but do represent the most frequent form of genital mutilation (61%) [1]. Most self-inflicted testicular injuries have been reported in transsexual patients who desire emasculation or by psychotic patients with either functional or organic brain diseases like schizophrenia or a severe personality disorder [2,3].

Amphetamine use [4] and cocaine use [5] have been associated with severe self-injurious behavior. To the best of our knowledge self-castration engendered by cannabis abuse has never been reported.

We report an uncommon case of a man with self-castration resulting from cannabis addiction.

### Case Presentation

**Figure 1:**
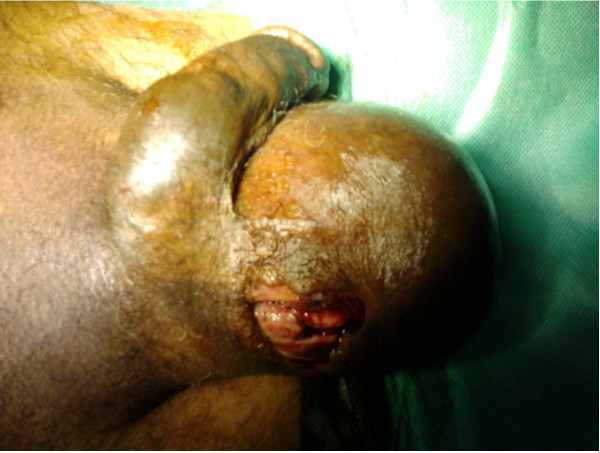
**Unilateral scrotal laceration**.

**Figure 2:**
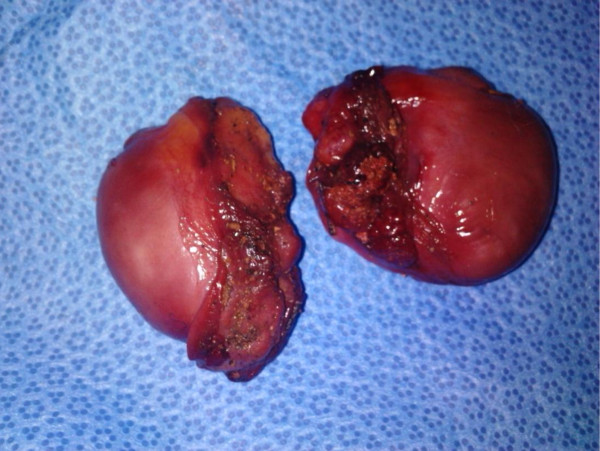
**Both testis after externalization**.

**Figure 3:**
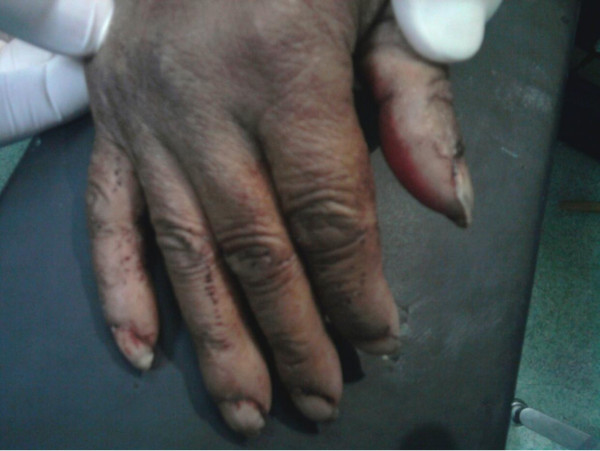
**Long fingernails**.

**Figure 4:**
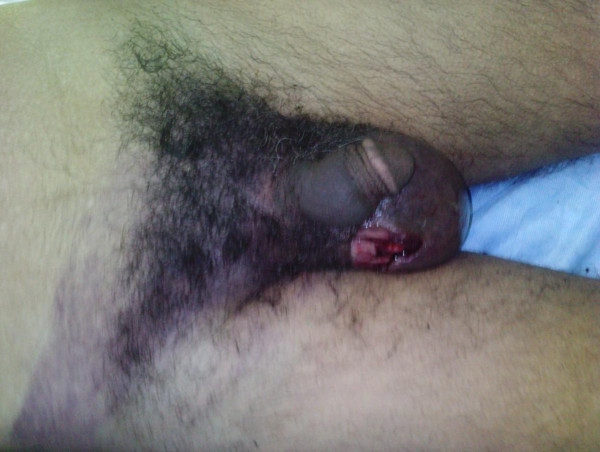
**Ecchymosis skin extended from his scrotum to the inguinal region**.

**Figure 5:**
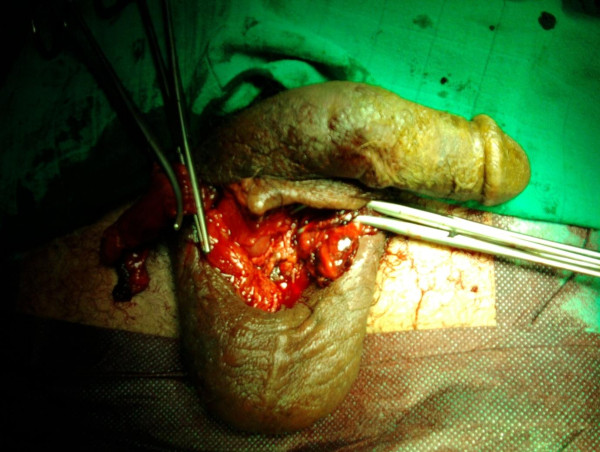
**Ligation of both spermatic cords**.

We describe the case of a 40-year-old Berber man, originally from Morocco, who presented to our emergency room with self-inflicted testicular injuries. His medical history was marked by tuberculosis of the lung. His psychiatric history dated from 32 years of age, when he was treated due to alcohol and cannabis abuse. Many medical treatments and psychotherapy techniques were proposed for detoxification but they failed because of his poor compliance with therapy. At the time of admission, he had not consumed alcohol for several months, but he reported using cannabis, particularly a few hours before the act. No childhood trauma, personality or even borderline personality disorders (assessed as a lifetime diagnosis) were diagnosed. He reported no psychiatric or medical diseases among close relatives. He presented to our emergency room eight hours later with unilateral scrotal laceration (Figure 1) and externalization of both testis (Figure 2) using his long fingersnails (Figure 3). This was associated with hemodynamic shock. Our patient underwent vascular filling and blood transfusion to achieve stabilization of his state. Upon examination of our patient's perineum, one wound was visible on the top of his right hemiscrotum measuring 4 cm with ecchymosis extended from his scrotum to the inguinal region. There was increase in scrotal volume due to the hematoma, with no active hemorrhage found (Figure 4). Our patient was interviewed by our psychiatrist, who found appropriate orientation and good contact. His speech was normal, with emotional indifference. His mood was mildly depressed, following the events that led to his admission. He reported no suicidal ideation, suicidal behavior, or desire for self-injury, and had no psychotic ideation. Computed tomography with intravenous contrast was performed to localize his spermatic cord, which was fortunately not retracted into the inguinal or retroperitoneal region. After stabilization of his psychiatric state with a benzodiazepine drug (diazepam: 30 drops a day), our patient was admitted to the operating room and haemostasis was easily achieved after ligation of both spermatic cords (Figure 5). The dartos and skin were then closed in two layers, with a good postoperative result. Our patient was discharged after his second day to a psychiatric department for supplement care and for substitution therapy for his cannabis use.

## Discussion

Self-mutilation, a very unusual situation in routine urology, is a direct and deliberate harm to one's body without conscious intent to die. It is observed in both men and women with various psychiatric disorders [6]. Self-castration is an uncommon phenomenon. It usually occurs in the context of a psychotic disorder, specifically schizophrenia [7], with evidence suggesting an increased prevalence of psychosis surrounding the time of self-castration [2,8]. Genital self-mutilation has also been documented in other patient populations, including individuals suffering from character pathology [7], substance abuse [4], gender identity issues [9], issues of religious content, guilt, sexual conflict, and with a history of depression with a severe suicide attempt, severe childhood deprivation, loss of a father and sexual identity disturbances specific to males [8]. Few cases have been reported within the last 20 years.

We report here the first case of a patient who self-mutilated his testes with his long fingernails under the influence of cannabis. Many theories consider self-mutilation to be a strategy to reduce distress or tension, an expression of anger or shame, or manipulative behavior. Some authors link this behavior to borderline personality disorder [10] or treat it as a means for the patient of controlling traumatic childhood experiences [11]. Our patient, however, had no history of childhood trauma or any axis II disorder. A high consumption of cannabis just before his act led us to the belief that cannabis abuse was the trigger for testicular self-mutilation. Self-mutilation may also be linked to difficulties in impulse control, as here. In any case, the clinical characteristics of self-mutilation are manifold, and its etiology is a topic for debate [12].

Cannabis, also known as "marijuana", "marihuana", "hashish" and "ganja", is a psychoactive drug, which is forbidden in many states. It is very prevalent in Africa, especially in Morocco, and in South America. There is a close relationship between dopamine and self-mutilation. High doses of dopaminergic agonists, such as amphetamine, can engender self-mutilation. We know that psychoactive substances (such as cocaine and cannabis) alter synaptic transmission by interacting with dopamine transporters, and that their dopaminergic action is one of their most important neurobiological properties. Gorea and Lombard report that the dopaminergic system may participate in mutilating behavior in rats [13].

The complex behavior associated with cannabis abuse may be one cause of self-mutilation. In animals, delta-9-tetrahydrocannabinol enhances dopaminergic neurotransmission in brain regions known to be implicated in psychosis. Studies in humans show that genetic vulnerability may add to increased risk of developing psychosis and cognitive impairments following cannabis consumption. Delta-9-tetrahydrocannabinol induces psychotic like states and memory impairments in healthy volunteers [14]. Dysfunction of the inhibitory brain circuitry in drug addiction [15] could explain why this patient lost control and mutilated himself following drug use.

Treatment for this patient population can be challenging. An integrated liaison-type psychiatric intervention can be effective in improving compliance with psychiatric treatment, surgical outcomes and reducing medical consumption [16]. The first step in the treatment of our patient was to admit to the surgical unit to achieve haemostasis. We had to first perform computed tomography with intravenous contrast to locate his spermatic cord, as our choice of incision depended on its location. If the spermatic cord is not retracted, a scrotal incision should be made, but in cases where the spermatic cord is not visible, then inguinal or retroperitoneal exploration should be attempted to gain access to the testicular vessels and provide hemostasis. Secondly, evidence has implicated serotonergic depletion and dopaminergic stimulation in self-injurious behaviors, supporting the use of paroxetine and risperidone, respectively, in this case [17,18]. Some authors have authorized the use of mood stabilizers such as lithium, valproic acid, or carbamazepine as alternative treatments. The role of psychotherapy can be effective for these patients in establishing a therapeutic alliance with a care provider and providing ego support.

Lastly, hormone replacement therapy based on testosterone was proposed to the patient and his family (with different pharmaceutical presentations: intramuscular, oral, patch). The risks of not treating this castration state were illustrated, with major risks being cardiovascular and osteoporotic, and other minor risks including asthenia, obesity and mood disorder.

## Conclusion

Self-mutilation behavior is increasingly observed in emergency departments, but the relationship between genital injuries and substance addiction, particularly cannabis abuse, has to the best of our knowledge never been described. This case report is therefore interesting and can lead to new investigation in this area.

## Consent

Written informed consent was obtained from the patient for publication of this manuscript and accompanying images. A copy of the written consent is available for review by the Editor-in-Chief of this journal.

## Competing interests

The authors declare that they have no competing interests.

## Authors' contributions

MA was the principal author and a major contributor in writing the manuscript. MFT analyzed and interpreted the patient data and review of the literature. AK, MJE, MHF read and corrected the manuscript. AB and KL both provided medical and surgical support for this case, and contributed to the writing of the paper. RSW contributed to the writing of the paper. All authors read and approved the final manuscript.
